# Supratentorial cerebrospinal fluid diversion using image-guided trigonal ventriculostomy during retrosigmoid craniotomy for cerebellopontine angle tumors

**DOI:** 10.3389/fsurg.2023.1198837

**Published:** 2023-05-23

**Authors:** Michel Roethlisberger, Noëmi Elisabeth Eberhard, Jonathan Rychen, Saif Al-Zahid, Ronie Romelean Jayapalan, Christian Zweifel, Ravindran Karuppiah, Vicknes Waran

**Affiliations:** ^1^ Department of Neurosurgery, University Hospital Basel, University of Basel, Basel, Switzerland; ^2^ Faculty of Medicine, University of Basel, Basel, Switzerland; ^3^ Department of Surgery, Division of Neurosurgery, University Malaya, Jalan Universiti, Kuala Lumpur, Malaysia; ^4^ Department of Otorhinolaryngology (ORL), University Malaya Specialist Centre, University of Malaya, Jalan Universiti, Kuala Lumpur, Malaysia; ^5^ Department of Otorhinolaryngology, Hereford County Hospital, Hereford, United Kingdom; ^6^ Department of Neurosurgery, Cantonal Hospital Graubuenden, Chur, Graubuenden, Switzerland

**Keywords:** ventriculostomy, cerebrospinal fluid diversion, trigonal, retrosigmoid craniotomy, cerebellopontine angle, image guided surgery, hemorrhage, upward transtentorial herniation

## Abstract

**Background:**

Cerebellar contusion, swelling and herniation is frequently encoutered upon durotomy in patients undergoing retrosigmoid craniotomy for cerebellopontine angle (CPA) tumors, despite using standard methods to obtain adequate cerebellar relaxation.

**Objective:**

The aim of this study is to report an alternative cerebrospinal fluid (CSF)-diversion method using image-guided ipsilateral trigonal ventriculostomy.

**Methods:**

Single-center retro- and prospective cohort study of *n* = 62 patients undergoing above-mentioned technique. Prior durotomy, CSF-diversion was performed to the point where the posterior fossa dura was visibly pulsatile. Outcome assessment consisted of the surgeon's intra- and postoperative clinical observations, and postoperative radiological imaging.

**Results:**

Fifty-two out of *n* = 62 (84%) cases were eligible for analysis. The surgeons consistently reported successful ventricular puncture and a pulsatile dura prior durotomy without cerebellar contusion, swelling or herniation through the dural incision in *n* = 51/52 (98%) cases. Forty-nine out of *n* = 52 (94%) catheters were placed correctly within the first attempt, with the majority of catheter tips (*n* = 50, 96%) located intraventricularly (grade 1 or 2). In *n* = 4/52 (8%) patients, postoperative imaging revealed evidence of a ventriculostomy-related hemorrhage (VRH) associated with an intracerebral hemorrhage [*n* = 2/52 (4%)] or an isolated intraventricular hemorrhage [*n* = 2/52 (4%)]. However, these hemorrhagic complications were not associated with neurological symptoms, surgical interventions or postoperative hydrocephalus. None of the evaluated patients demonstrated radiological signs of upward transtentorial herniation.

**Conclusion:**

The method described above efficiently allows CSF-diversion prior durotomy to reduce cerebellar pressure during retrosigmoid approach for CPA tumors. However, there is an inherent risk of subclinical supratentorial hemorrhagic complications.

## Introduction

Surgery for tumors of the cerebellopontine angle (CPA) requires meticulous care of the cerebellar cortex, which is known to be softer than the cerebral cortex (1). A higher resistance of the venous outflow caused by the mass lesion and the patient's positioning potentially leads to a raised pressure within the posterior fossa. Preoperative measures to reduce said complications are temporary cerebrospinal fluid (CSF)-diversion with a lumbar drain, and the semi-sitting or sitting position of the patient, however, each with their own spectrum of advantages and disadvantages. Standard methods of obtaining adequate cerebellar relaxation during surgery are anesthesiologic management and the microsurgical puncture of arachnoid cisterns or cerebellar fissures (2–4). In certain cases, however, the surgeon encouters cerebellar contusion, swelling and herniation upon durotomy in which the release of CSF remains difficult (2, 5). Excessive retraction maneuvers cause tissue trauma, and the resulting cortical contusions promote further cerebellar swelling (2, 6). All of the above-mentioned measures have their own risk-benefit profile, potentially making it unnecessary to perform a supratentorial ventriculostomy. To further widen the surgical armamentarium and evaluate the safety of such a reserve strategy, the authors used a supratentorial CSF-diversion method using image-guided ipsilateral trigonal ventriculostomy to reduce cerebellar pressure prior to durotomy when resecting CPA-tumors via the retrosigmoid approach (7, 8). To date, no cohort study determining this methods accuracy and outcome has been reported in the English literature. The aim of this study is to report technical nuances of the above-mentioned technique, and to report surgical outcomes and complications in a retrospectively and prospectively collected consecutive single-center cohort.

## Methods

The data that support the findings of this study are reported according to the STROBE guidelines and are available on reasonable request (9).

### Registration

Local ethical committee approval was obtained from the last authors' institution (MREC ID NO: 2017127-4864) and the requirement for written informed consent was waived (justification: disproportionality). No clinical trial registration was required.

### Study design

Single-center case series based on data collected between March 2012 and May 2019 (retrospective 2012–2017 and prospective 2018–2019).

### Participants and study setting

**Figure 1:**
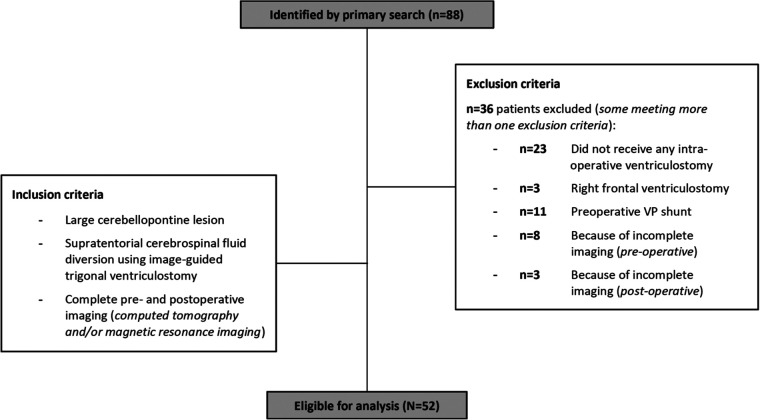
Patient inclusion profile: Patients, where standard methods of CSF-release without a ventriculostomy (*n* = 23) or a ventriculostomy via a right frontal trajectory (*n* = 3) was used were excluded from the study, resulting in *n* = 62 eligible patients. Additionally, patients with a pre-existing permanent CSF-diversion (*n* = 11), and patients with missing information on pre- (*n* = 8) or postoperative imaging (*n* = 3), were excluded. Certain patients met more than one exclusion criteria. Eventually, *n* = 52 patients were included into the final analysis.

Patients undergoing supratentorial CSF-diversion using image-guided trigonal ventriculostomy prior to retrosigmoid craniotomy for the resection of a CPA-tumor, irrespective of the ventricular size on preoperative imaging (Figure 1). To minimize selection bias within the reported cohort, the technique was used within a consecutive series patients and pathologies (Table 1). Regular follow-up consisted of clinical postoperative intermediate care visits and outpatient follow-up performed by the last author 4–6 weeks after surgery and then once per year.

**Table 1:** Baseline characteristics of *n* = 52 patients recieving supratentorial cerebrospinal fluid diversion using image-guided trigonal ventriculostomy during retrosigmoid craniotomy for cerebellopontine angle tumors.

Patient characteristics	Total (*n* = 52, 100%)
**Sex**	
Female [*n* (%)]	31 [59.6]
Female/male ratio	1.47
**Age**	
Mean age in years [median (range)]	53 [55 (11–79)]
**Tumor characteristics**	
**Side**	
Left [*n* (%)]	24 (46%)
Right/left ratio	1.17
**Preoperative tumor volume**	
Mean volume in cm^3^ [median (range)]^a^	22 [15; (4–88)]
**Localization**	
Cerebellopontine angle	40 (77%)
Spheno-petroclival	5 (10%)
Petrous apex	4 (8%)
Tentorium	2 (4%)
Foramen magnum	1 (2%)
**Histology**	
Vestibular schwannoma	29 (62%)
Meningioma	14 (27%)
Trigeminal schwannoma	3 (6%)
Lower cranial nerve schwannoma	2 (4%)
Peripheral nerve sheet tumor	1 (2%)
Cholesterol granuloma	1 (2%)
Epidermoid	1 (2%)
Primitive neuro-ectodermal tumor	1 (2%)
**Koos Grade in vestibular schwannoma (*n* = 29)**	
I or II	0 (0%)
III	8 (28%)
IV	21 (73%)
**Volumetrics**	
Mean posterior fossa volume cm^3^ [median (range)]^b^	176 [179 (131–222)]
Mean tumor volume / posterior fossa volume ratio	0.125
**Hydrocephalus**	**14 (27%)**
**Affection of 4th Ventricle**	
Open	13 (25%)
Distorted but patent	32 (61%)
Obstructed	7 (14%)
**Brainstem configuration**	
Normal	3 (6%)
Impression	17 (33%)
Compression	22 (42%)
Dislocation	10 (19%)
**Cerebellar Edema**	
None	41 (79%)
Unilateral	9 (17%)
Bilateral	2 (4%)
**Tonsillar transforaminal herniation**	**7 (14%)**
**Effacement of CSF cisterns surrounding the brainstem**	**18 (35%)**
**Radiological signs of upward transtentorial herniation**	
Upward transtentorial herniation	1 (2%)
Flattening or reversal of the smile-shaped quadrigeminal cistern	12 (23%)
Obliteration of the quadrigeminal and superior cerebellar cistern	8 (15%)
“Spinning top” appearance of midbrain^c^	1 (2%)

CPA, cerebellopontine angle; CSF, cerebrospinal fluid.

^a^ The volume of the tumor was contoured on each slice of the available MR and CT scans.

^b^ The volume of the posterior fossa was contoured from the tentorial opening, along the tentorial border and the bony border of cerebellar hemispheres down to the foramen magnum.

^c^ Due to bilateral compression of the posterior aspect of the midbrain.

### Surgical technique

**Figure 2:**
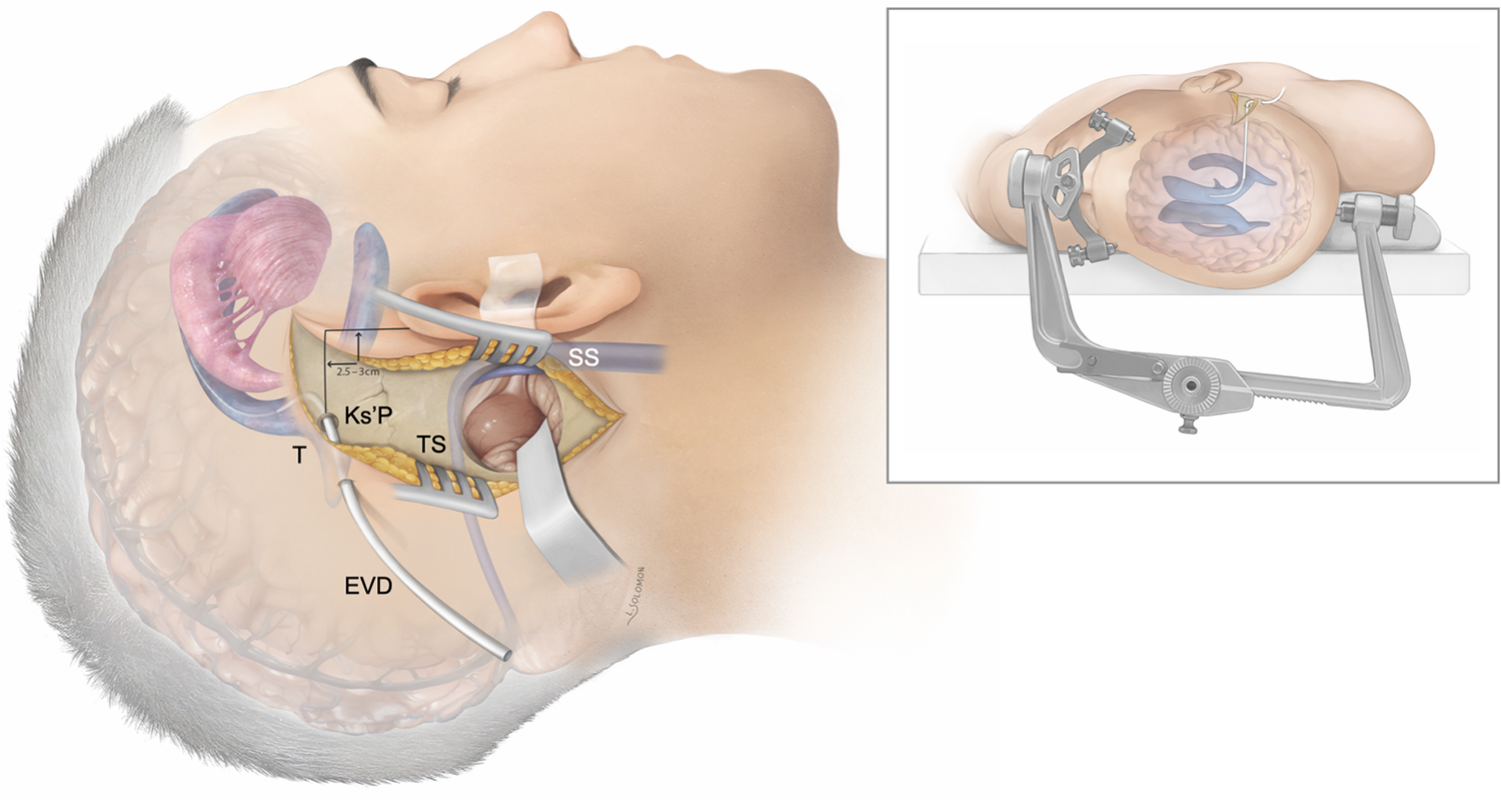
Patient positioning and general concept: Artistic rendition of the supratentorial cerebrospinal fluid diversion method using image-guided trigonal (T) ventriculostomy via the Keen’s point (Ks’P) to achieve cerebellar relaxation during retrosigmoid craniotomy for cerebellopontine angle tumors. The patient is in a supine position and the head fixated in rotation and slightly latero-flexed to the contralateral side using a standard Mayfield clamp. Transverse sinus (TS); sigmoid sinus (SS).

**Figure 3:**
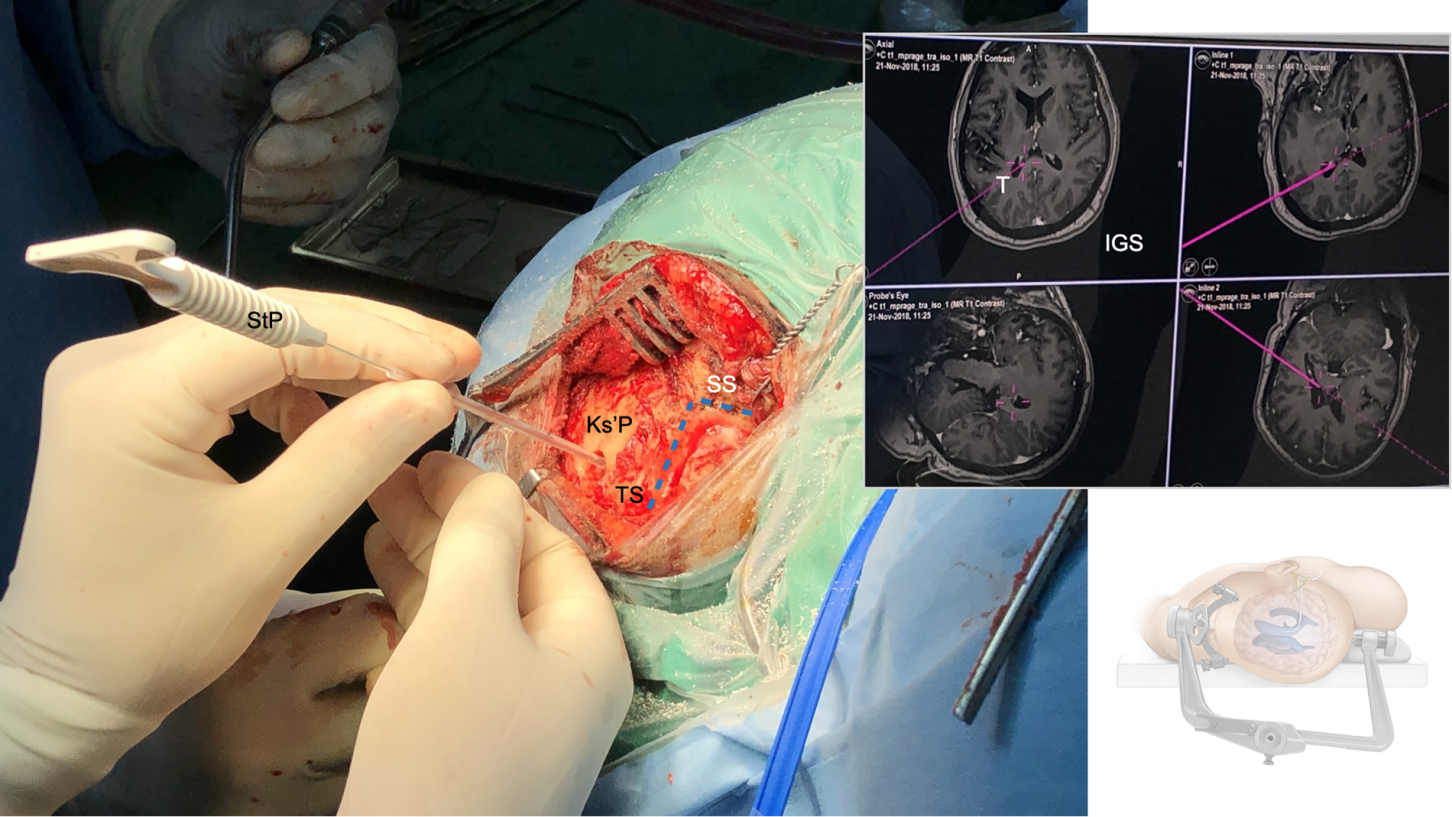
Supratentorial image-guided trigonal ventriculostomy: After myocutaneous skin incision, the course (blue lines) of the transverse (TS) and sigmoid sinus (SS) and the transverso-sigmoid junction (TSJ) are defined superficially along the bony surface. A burr hole is placed with the trajectory aimed towards ipsilateral occipital horns of the lateral ventricle, the dura is opened and a small corticotomy is performed. A sterile image-guidance probe (StP) replacing the supply trocar of the external ventricular drain (EVD) is inserted into the ipsilateral occipital horn of the lateral ventricle (usually with a loss of resistance after 4–5 cm), aimed in a slight cephalic direction, and under constant image-guidance (IGS). The ventricular catheter is further advanced in soft pass technique after positive CSF-outflow out of the catheter (usually up to 6–7 cm).

**Figure 4:**
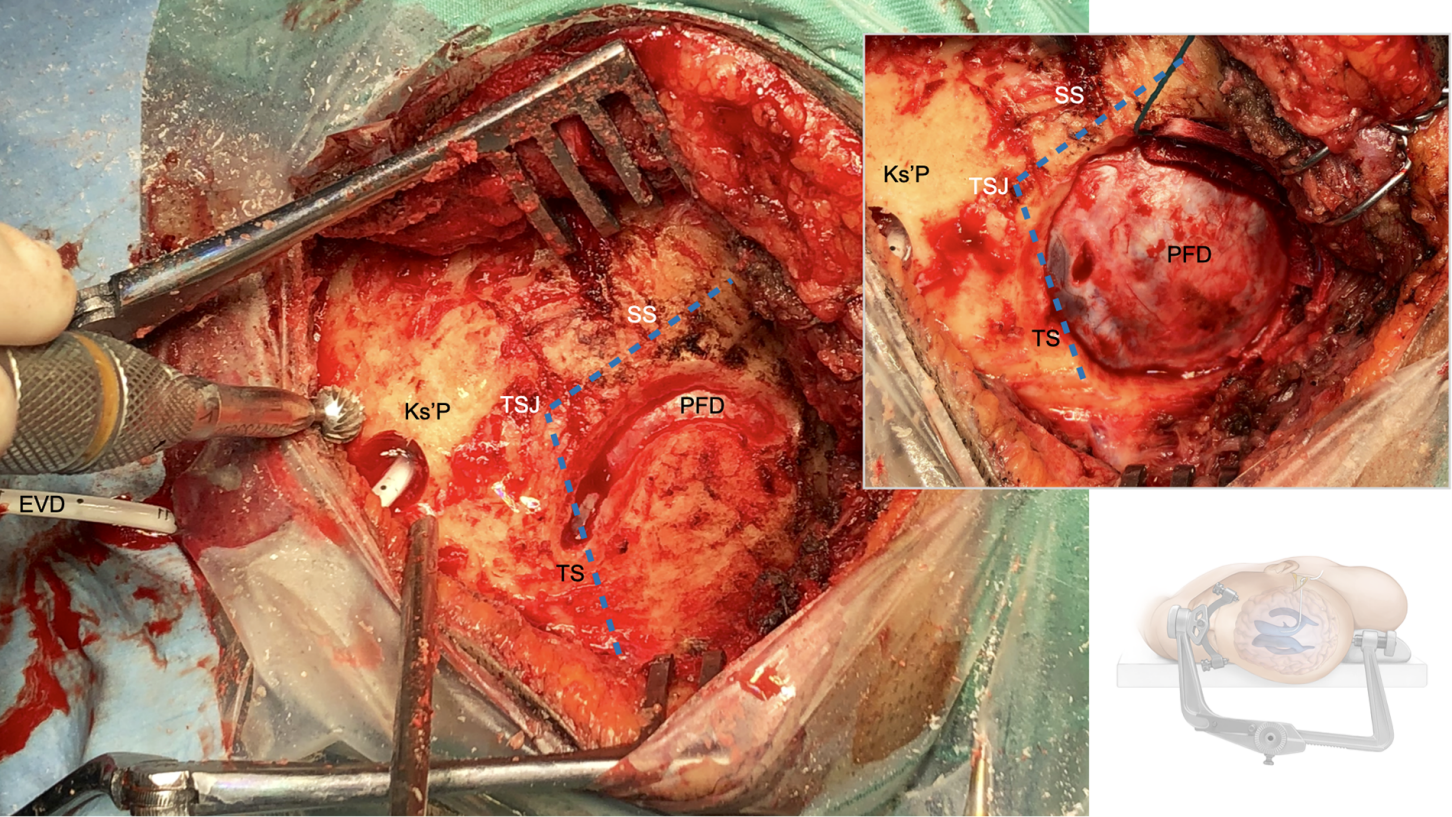
Retrosigmoid craniotomy: The transverse (TS) and the sigmoid (SS) sinus as well as the transverso-sigmoid junction (TSJ) are dlineated and skeletonized. The bone flap is then raised using a craniotome and the posterior fossa dura (PFD) is visualized. The external ventricular drain (EVD) is then tunneled from the Keen's point (Ks'P) out about 5 cm away from the surgical field.

The patients are positioned supine with their head rotated and slightly latero-flexed to the contralateral side. Image-guided surgery (IGS) is used based on preoperative computed tomography (CT) or magnetic resonance imaging (MRI) scans. Following a superiorly extended post-auricular “C” shaped incision and delineation of the dural sinuses, a standard retrosigmoid craniotomy is performed. A burr hole is then placed based on IGS to be included within primary incision approximately 2.5 cm superior and posterior to the pinna of the ear. Prior to durotomy, an image-guided ventriculostomy of the ipsilateral trigone of the lateral ventricle is performed using a sterile image guidance probe aimed in a slight cephalic direction, and the external ventricular drain (EVD) is advanced 4–5 cm perpendicular to the cortex as it penetrates the proximal wall of the ventricle (7, 8). A loss of resistance is usually felt after 4–5 cm, and from that point, the catheter is advanced in soft pass technique after removing the image guidance probe. In most of the cases, there is some CSF flow out of the ventriculostomy track and CSF flow is observed while the ventricular catheter is softly advanced (usually 6–7 cm). The amount of CSF released is performed to the point where the posterior fossa dura is visibly pulsatile. The EVD is then tunneled out about 2–5 cm away from the surgical field. Following a standard durotomy, cerebellar relaxation was confirmed in cases where the brain has spontaeously fallen off the dura (Figures 2–4 and Supplementary Video S1).

### Variables and definitions

*Baseline variables*: patient characteristics; initial tumor characteristics [volume (cm^3^), morphology, pathology]; posterior fossa characteristics (volume [cm^3^] and tumor/posterior fossa ratio (10); volumetric analysis of the preoperative tumor volume, the posterior fossa and their ratio was performed on preoperative MRI sequences (T1-Gd, T2) using a volume rendering software (BrainLab AG. Release date 2013. iPlan® Cranial, Version 3.0. Feldkirchen, Germany) (11); affection of the fourth ventricle (open, distorted but patent or obstructed) and brainstem (normal, impressed, compressed or dislocated) (12); preoperative tonsillar transforaminal herniation (effacement of the CSF-cisterns surrounding the brainstem, inferior descent of the cerebellar tonsils below the level of the foramen magnum); *Surgical outcome variables*: relaxation of the surgical field upon durotomy: intraoperative assessment on successful ventricle puncture, CSF-diversion and a pulsatile dura prior durotomy (Supplementary Video S1) without signs of cerebellar contusion, swelling or herniation through the dural incision. To reduce selection bias, a consecutive series of patients irrespective of the underlying pathology was included where the last author (VW) was involved in the surgical procedure; pre- or postoperative hydrocephalus (malresorptive or obstructive) based on clinical and radiological examination, pre- or postoperative permanent CSF-diversion; *Radiographic outcome variables*: Catheter malposition, defined as grade 1 (ipsilateral intraventricular), grade 2 (contralateral intraventricular), grade 3 (parenchymal or deep/eloquent areas) (13, 14); ventriculostomy-related hemorrhage (VRH); intracerebral hemorrhage; intraventricular hemorrhage; cortical cortical scarring along the ventriculostomy canal; cortical atrophy and scaring caused by hemorrhage; signs of infarction; pre- and postoperative signs of upward transtentorial herniation (1. flattening or reversal of the smile-shaped quadrigeminal cistern; 2. obliteration of the quadrigeminal and superior cerebellar cistern; 3. “spinning top” appearance of midbrain due to bilateral compression of the posterior aspect of the midbrain) (15, 16); posterior cerebral or superior cerebellar artery infarction. *Clinical outcome variables*: Ventriculostomy-related infections (VRI), ventriculostomy-related seizures and clinically relevant ventriculostomy-related visual field defects in the follow-up examinations (14).

### Statistical considerations

For descriptive analyses, we report medians and ranges for continuous variables, and percentages for categorical variables. The primary endpoint of the study was the detection of any supratentorial hemorrhagic complication associated with the ventriculostomy in postoperative imaging. The secondary endpoints were signs of upward transtentorial herniation and the frequency of postoperative hydrocephalus. To obtain associations between the primary and secondary endpoints, the binary logistic regression model was used. Risk factors that had more than 10% increase or decrease in OR (OR ≤ 0.9 or ≥1.10) and which were considered clinically relevant for the endpoints of interest (Supplementary Table S1) were included in the multivariable model. Confidence intervals were calculated with the profile likelihood method based on the Wald test statistic. Statistical significance was set at *p* ≤ .05. Statistical analysis was performed using SPSS [IBM SPSS Statistics 28.0.1.0 (142), 2013, New York, USA].

## Results

### Baseline characteristics

Fifty-two out of *n* = 62 cases (84%) were eligible for analysis (Figure 1). Mean patient age was 53 (range 11–79 years). The cohort consisted of *n* = 34/52 (65%) cranial nerve schwannoma's, *n* = 14/52 (27%) meningioma's, and *n* = 4/52 (8%) rare pathologies of the CPA. Fourteen out of *n* = 52 patients (27%) suffered from preoperative obstructive hydrocephalus. 4th ventricle compression or obstruction was present in *n* = 39/52 (75%), and brainstem compression or dislocation in *n* = 32/52 (62%) cases. Radiological signs of tonsillar transforaminal herniation were found in *n* = 7/52 cases (14%), and cerebellar upward transtentorial herniation only in one single case (2%) that met the predefined radiological criteria (Table 1) (15, 16).

### Surgical outcomes

#### Intraoperative outcomes

Successful ventricle puncture and a pulsatile dura prior durotomy without cerebellar contusion, swelling or herniation through the dural incision was consistently reported except for one case (2%) in a very short necked patient with a medial third petroclival meningioma, where protracted and uncontrollable cerebellar swelling occurred throughout the whole procedure. No failed punctures were recorded, with *n* = 49/52 (94%) correctly placed EVD's on the first, and *n* = 3/52 (6%) on the second attempt. The postoperatively closed EVD was removed in *n* = 40/52 patients (77%) within 48 h after surgery. Twelve out of *n* = 52 patients (23%) needed prolonged EVD weaning because of postoperative clinical and/or radiological signs of hydrocephalus. Eventually, *n* = 5/52 patients (10%) needed a permanent CSF-diversion in the longer term (mean 91 days, range 3–246) (Table 2).

**Table 2:** Surgical, radiographic and clinical outcome variables of n = 52 patients patients recieving supratentorial cerebrospinal fluid diversion using image-guided trigonal ventriculostomy during retrosigmoid craniotomy for cerebellopontine angle tumors.

Surgical outcomes	Total (*n* = 52, 100%)
Single attempt for successful ventriculostomy	49 (94%)
Two attempts for successful ventriculostomy	3 (6%)
Successful CSF-diversion and pulsatile dura prior durotomy without signs of cerebellar contusion, swelling or herniation through the dural incision	51 (98%)
**Postoperative hydrocephalus**	
Due to impaired resorption	4 (8%)
Due to 4th ventricle compression	6 (11%)
Due to upward transtentorial herniation	2 (4%)
Permanent CSF-diversion after surgery	5 (11%)
Days to shunting [mean, (min-max), median]	91, [3–246], 30
**Radiographic outcomes**	
**Catheter tip location and grade**	
Grade 1: Ipsilateral trigonum, occipital horn or lateral ventricle	35 (67%)
Grade 2: Contralateral trigonum, occipital horn or lateral ventricle / interhemispheric fissure	15 (28%)
Grade 3: Intraparenchymal or basal ganglia	2 (4%)
**Supratentorial hemorrhagic complications**	
Ventriculostomy-related hemorrhage	6 (11%)
Intracerebral hemorrhage	2 (4%)
Intraventricular hemorrhage	2 (4%)
Midline shift	1 (2%)
Ventriculostomy-related revision surgery	0 (0%)
**Parenchymal lesion in magnetic resonance imaging**	
One visible catheter tract	41 (79%)
Two visible catheter tracts	3 (6%)
No visible ventriculostomy tract	8 (15%)
Cortical scarring related to ventriculostomy	15 (29%)
Signs of local infarction	1 (2%)
PCA/SCA infarction	0 (0%)
Radiological signs of upward trans-tentorial herniation	0 (0%)
**Clinical outcomes**	
Seizures related to supratentorial ventriculostomy-related hemorrhage	0 (0%)
Ventriculostomy-related homonymous hemianopsia	0 (0%)
Ventriculostomy-related focal neurological deficit	0 (0%)
Ventriculostomy-related infection	0 (0%)

MRI, magnetic resonance imaging; CSF, cerebrospinal fluid; PCA, posterior cerebral artery; SCA, superior cerebellar artery.

#### Radiographic complications

**Figure 5:**
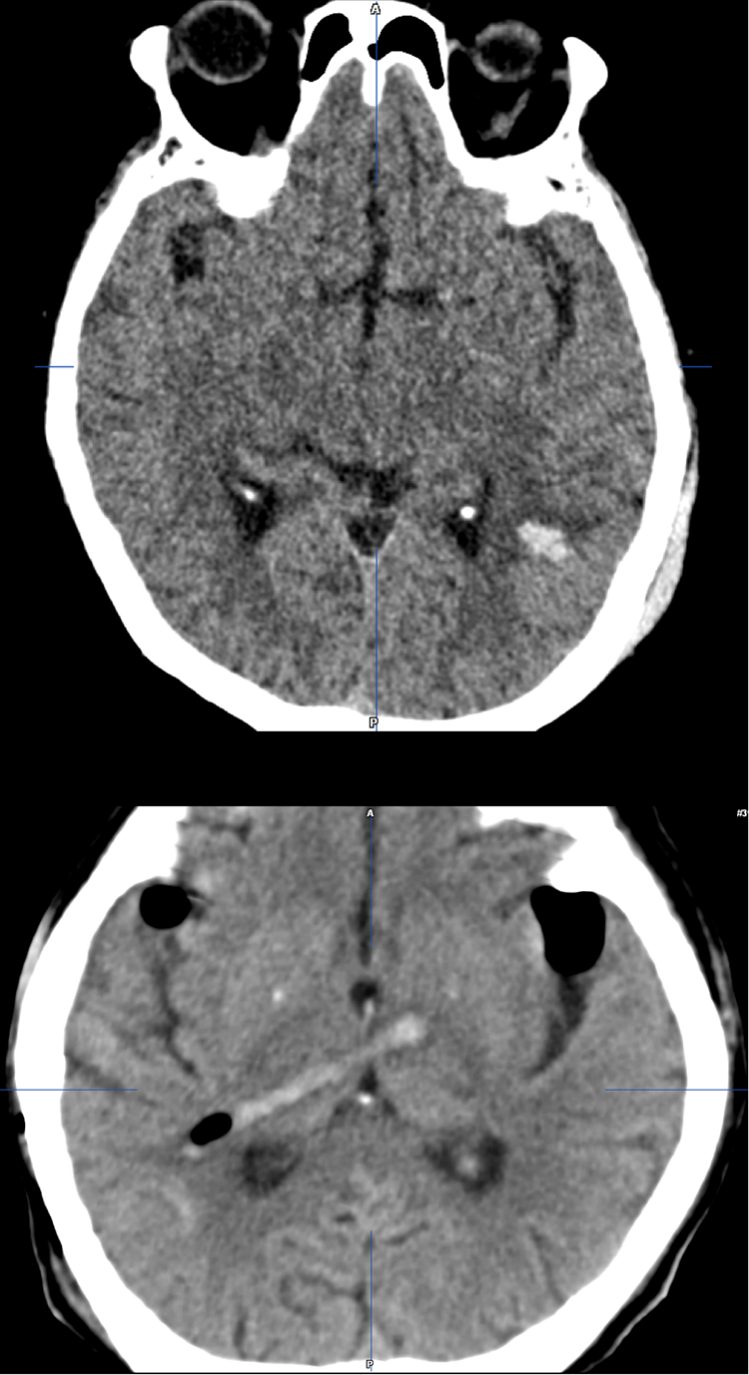
Ventriculostomy-related supratentorial complications: ventriculostomy-related intracerebral hemorrhage in the left parieto-occipital parenchyma within the area of the trigonal ventriculostomy [upper]; ventriculostomy-related hemorrhage along the catheter track; grade 3 malposition (catheter through parenchyma and tip position in the basal ganglia) [lower].

No significant signs of cerebellar contusion or swelling were noted in the post-operative scans. In the majority of patients (*n* = 50, 96%), the catheter tip was intraventricular (grade 1 or 2) except for two cases (4%), where the catheter tip was found intraparenchymal (e.g., basal ganglia) (13, 14). In *n* = 4/52 patients (8%), evidence of a VRH in association with an intracerebral hemorrhage [*n* = 2/52 (4%)] or an isolated intraventricular hemorrhage [*n* = 2/52 (4%)] was found in the postoperative imaging. In most of the patients, a ventriculostomy track was visible in the postoperative follow-up MRI. A supratentorial cortical scar along the ventriculostomy canal was seen in *n* = 15/52 cases (29%). None of the included patients showed radiological signs of upward transtentorial herniation postoperatively (Table 2 and Figure 5). Uni- and multivariable regression analysis did not detect any association of VRH, neither with one of the relevant baseline variables, postoperative hydrocephalus nor permanent CSF-diversion (Table 3 and Supplementary Table S1).

**Table 3:** Multivariable covariate binary logistic regression analysis on risk-factors associated with postoperative hydrocephalus; any supratentorial hemorrhagic complication (including ventriculostomy-related, intraventricular and intracerebral hemorrhage); and permanent CSF-diversion in *n* = 52 patients undergoing supratentorial CSF-diversion using image-guided trigonal ventriculostomy during retrosigmoid craniotomy for CPA-tumors.

Multivariable analysis				
Postoperative hydrocephalus	OR	95% CI		*p*-value
		Lower	Upper	
Age ≥60 years	2.69	0.18	41.2	0.476
Female sex	0.02	0.00	0.37	0.008**
Preoperative hydrocephalus	60.2	3.25	11.1	0.006**
Preoperative 4th ventricle affection	0.41	0.03	6.34	0.525
Preoperative brainstem affection	4.30	0.33	56.0	0.265
Preoperative cerebellar edema	2.64	0.43	16.2	0.294
Preoperative tonsillar herniation	0.05	0.00	1.63	0.093
Supratentorial hemorrhagic complication	2.22	0.12	40.5	0.590
Supratentorial hemorrhagic complication	OR	95% CI		*p*-value
		Lower	Upper	
Age ≥60 years	1.81	0.36	9.17	0.471
Female sex	2.95	0.47	18.7	0.251
Preoperative hydrocephalus	0.79	0.10	6.04	0.823
Preoperative 4th ventricle affection	4.47	0.42	47.4	0.213
Preoperative brainstem affection	4.55	0.46	44.5	0.193
Preoperative cerebellar edema	0.35	0.04	3.33	0.362
Preoperative tonsillar herniation	0.63	0.04	9.21	0.733
Permanent CSF-diversion after retrosigmoid craniotomy	OR	95% CI		*p*-value
		Lower	Upper	
Age (per year)	0.92	0.86	0.99	0.022*
Preoperative Hydrocephalus	2.12	0.19	24.0	0.546
Preoperative 4th ventricle affection	0.62	0.01	6.64	0.416
Preoperative brainstem affection	3.25	0.21	49.1	0.398
Preoperative cerebellar edema	1.04	0.17	6.50	0.970
Preoperative tonsillar herniation	2.75	0.14	54.7	0.508

CSF, cerebro-spinal fluid; OR, Odds-ratio; CI, confidence-interval.

Male sex and preoperative hydrocephalus were associated with postoperative hydrocephalus. The risk for a permanent CSF-diversion procedure was found to be less likely in younger patients (8% less risk per additional year). The binary logistic regression model includes the following covariates: age ≥60 years (reference level: age <60 years), age (continuous per year), female sex (reference level: male sex), posterior fossa volume (cm^3^), tumor volume (cm^3^) and posterior fossa / tumor volume ratio (continuous), preoperative hydrocephalus (reference level: absence of hydrocephalus), preoperative 4th ventricular compression / obstruction (reference level: absence of 4th ventricular affection), preoperative brainstem compression / dislocation (reference level: absence of brainstem affection), preoperative cerebellar edema (reference level: absence of cerebellar edema), preoperative tonsillar herniation (reference level: absence of tonsillar herniation), and any supratentorial hemorrhagic complication consisting VRH, IVH and/or ICH (reference level: absence of supratentorial hemorrhagic complication); insignificance was set at alpha level of *p* = .05. Significance is indicated as follows: * (*p* ≤ .05), ** (*p* ≤ .01).

#### Clinical outcomes

Neither of the above-described hemorrhagic complications resulted in clinical symptoms including seizures or clinically relevant homonymous hemianopsia related to the cortical bleeding, or focal neurological deficits related to malpositioning of the catheters in the follow-up period (Table 2).

## Discussion

This cohort study reports the elective use of image-guided trigonal ventriculostomy during retrosigmoid craniotomy for CPA-tumors. We found a pulsatile dura prior durotomy without signs of cerebellar contusion, swelling or herniation through the dural opening in *n* = 51/52 (98%) cases, however, with an inherent risk of supratentorial hemorrhagic complications.

Brain relaxation has been defined by Li et al. as the relationship between the volume of intracranial contents and the capacity of the intracranial space upon durotomy (3). Hence, optimal brain relaxation is achieved when the volume of intracranial contents equals or is less than the capacity of the intracranial space, while inadequate brain relaxation is the result of an intracranial content volume surpassing this capacity (3). Optimal brain relaxation is a routinely assessed aspect of anesthetic care during neurosurgical procedures, leading to improved operating conditions and reduced necessity for brain retraction (3). This is of special importance when operating on large lesions of the CPA, where a higher resistance of the venous outflow and the patient's positioning leads to a raised pressure within the posterior fossa (3, 17). The importance of having cerebellar relaxation upon durotomy and non-traumatic retraction of the cerebellum to expose CPA-tumors cannot be over emphasized. The surgeon routinely evaluates how tight or relaxed the cerebellum is, or if swelling or even cerebellar herniation occurs. Standard maneuvers to soften the cerebellum and obtaining an optimal volume of the intracranial contents in relationship to the capacity of the intracranial space include anesthesiologic interventions and the microsurgical puncture of the horizontal fissure or the lateral cerebellopontine or cerebellomedullary cistern (1–3, 5). Release of CSF from the cisternal spaces is the commonest and most effective surgical maneuver, however, it can remain difficult when there is evident cerebellar compression and edema due to the posterior mass lesion or when hydrocephalus is present at the time of the dural opening. Heavy-handed retraction when attempting to open the basal cisterns can often lead to injury to the cerebellar hemisphere. Retraction and manipulation of the cerebellar cortex, known to be softer than the cerebral cortex, is unavoidable in these cases, and frequently results in retraction injuries or ischemia from compression (1, 3). Lumbar CSF-drains are often placed just prior to posterior fossa craniotomies to improve mean approach time to reach the cisterns, duration of hemostasis and to prevent CSF-leaks (18). However, the rate for moderate to severe complications associated with lumbar CSF-drains is not negligible, intraoperative obstruction occurs, and placement can be difficult in certain cases (19, 20). An expansion of the standard retrosigmoid craniotomy is a well-described option to avoid cerebellar retraction. Removal of the bone covering the sigmoid sinus allows its reflection and increases the angle of exposure to the cerebellopontine cistern by 50% while shortening the distance to the internal acoustic meatus (21). Additionally, a decreased cerebellar retraction pressure of 50%–60% was demonstrated (22). However, exposing and mobilizing the sigmoid sinus can lead to hemorrhagic and thrombotic complications (23). The retrosigmoid craniotomy can also be extended to the foramen magnum with exposure of the cerebellomedullary cistern. This approach overlaps the so called far lateral- or extended suboccipital approach and might be particularly helpful in tumors extending down to the foramen magnum (24). Cerebellar swelling can be prevented not only by extended exposure, but also with the optimal positioning of the patient. Our patients were operated in the supine position. It is likely that the venous pressure is raised in a supine position with the head rotated away, especially when compared to the semi-sitting or sitting position. The latter may be advantageous for larger masses in the posterior fossa, since the venous brain compartment is relieved and the brain is less prone to swelling, however, with the disadvantage of elaborate positioning and the inherent risk of air-embolism (4). Finally, the prophylactic placement of a frontal EVD or preoperative ventriculo-peritoneal shunt has been advocated if there is any concern in posterior fossa tenseness without obvious hydrocephalus, however, with the disadvantage of additional surgery and the potential risk of overtreatment (25).

The Keen's point was first described in 1890 for emergent CSF-diversion during posterior fossa surgery (7). The regular use of a supratentorial ventriculostomy to release CSF from the trigonum and achieve a relaxed field for large posterior fossa lesions was later described by Dandy in 1925 (26). The Keen's point, anatomically referring to the posterior parietal point, thus found common use for the elective proximal placing of ventriculoperitoneal shunt catheters, endoscopic evacuation of spontaneous intracerebral hemorrhage, and ventriculostomy for traumatic brain injury (7, 27–30). However, there is a lack of evidence on its accuracy, safety and radiological outcomes within a contemporary cohort of elective CPA-surgeries (7). The intraoperative integration of a trigonal ventriculostomy during retrosigmoid craniotomies requires a slightly larger incision, which does not seem to have any noticeable cosmetic effect on the patient (Figures 2–4). Both the burr hole for the EVD and the retrosigmoid craniotomy can be neatly contained within a single incision.

Freehand puncture of the ventricular system has inherent limitations in its accuracy and the potential for multiple attempts, thus carrying a higher risk of intracranial injury and hemorrhagic complications (14, 31, 32). Accurate placement after a primary freehand puncture of the frontal lateral ventricle is reported to be between 57%–91% (31–34), with a 10% rate of functional placement in the contralateral lateral ventricle or non-eloquent cortex, and an up to 13% rate of suboptimal placement in the eloquent cortex or nontarget cerebrospinal fluid space, with or without functional drainage (13). The given limitations of freehand trigonal puncture are even more relevant during retrosigmoid approaches, where the patients are positioned supine with the head being rotated away from the side of the lesion, a certain degree of neck flexion and the vertex oriented downwards. This position can be disorientating especially once the patient has been draped (Figures 2–4). Intraoperative image-guidance has significantly improved accuracy of catheter placement of ventricular catheters for temporary and permanent CSF-diversion (35–38). This is confirmed in our cohort study where only 3 out of 49 (6%) patients needed a 2nd attempt (Table 2). However, catheter localization errors can cause significant variations at the target and along the insertion trajectory, caused by anatomical differences between the image and the patient space or transformation errors of the surgical tool (39, 40). Our study confirmed a 28% (*n* = 15/52) rate of grade 2 (contralateral ventricular) and a 4% (*n* = 2/52) rate of grade 3 (parenchymal, eloquent) catheter malposition, being in line with a recently published contemporary multicenter register study on EVDs complications (Table 2 and Figure 5) (13, 14). In terms of trigonal ventriculostomy, not inserting the image-guidance probe more than 4–5 cm without a loss of resistance, and not advancing the catheter in soft pass technique more than 6–7 cm if there is no observable CSF-outflow, might reduce the risk of catheter malposition (Figures 2–4 and Supplementary Video S1).

Hemorrhagic and parenchymal injuries from cortical vessel damage are reported from 1% up to 20%–46% after supratentorial ventriculostomies. Known risk-factors include female sex, mean systolic blood pressure, amount of infused mannitol during anesthesia, smaller catheter diameter, antiplatelet intake and bed-side placement (34, 41–46). Hemorrhage is likely caused by cortical vein injury, explaining why neurological symptoms and severe complications by VRH remain extremely rare and are reported in only 0.4% of the affected patients (47, 48). Using trigonal ventriculostomy, VRH was reported in 7.7% of patients with aneurysmal subarachnoid hemorrhage prior to pterional craniotomy (49), and confirmed by our study on patients undergoing retrosigmoid craniotomy for the resection of a CPA-tumor, where 6/52 (11%) of patients were detected to have VRH without neurological symptoms or related complications (Table 2 and Figure 5).

Cerebellar upward transtentorial herniation was only detected in one patient (2%) preoperatively and is a well-known phenomenon in CPA-tumors (15, 50). The reported risk of clinical worsening of patients after ventriculostomy due to accelerated upward transtentorial herniation remains very low (51). In line with these results, none of our included patients showed radiological signs or sequelae of accelerated upward transtentorial herniation postoperatively (Table 2). Hydrocephalus requiring permanent CSF-diversion following CPA-surgery has been reported in up to 5%–7% for vestibular schwannoma (25, 52). Multivariable analysis revealed no association of hemorrhagic events with the 10% permanent CSF diversion rate in our case series (Tables 2, 3).

The current literature and our findings support, that supratentorial CSF-diversion should not be routinely used for smaller lesions, but only considered in large posterior fossa masses to facilitate resection after standard methods of obtaining adequate cerebellar relaxation remain insufficient.

### Limitations

A strength of the present cohort study lies in the homogeneity of the surgical procedure in a well-defined cohort with strict exclusion criteria. Some data were missing and could not be obtained despite all efforts, leading to exclusion of some patients. The safety of trigonal puncture for CSF-diversion is well established, thus, we did not perform systematic postoperative visual field examinations, but focused on clinically relevant homonymous hemianopsia in the postoperative follow-up period. The results are limited by the retrospective nature of the data and the single-center design, and larger studies are needed to confirm our results.

## Conclusion

Supratentorial CSF-diversion using image-guided trigonal ventriculostomy during retrosigmoid craniotomy for CPA-tumors efficiently allows to avoid cerebellar contusion, swelling or herniation through the dural incision. However, there is an inherent risk of subclinical supratentorial hemorrhagic complications.

## Previous presentation

The abstract of this study was presented as a poster at the 4th SFCNS Congress (Swiss Federation of Clinical Neuro-Societies) at the Swiss Tech Convention Center, EPFL (École Polytechnique Fédérale de Lausanne), Lausanne, Switzerland (10/25/2019); as a poster at the Virtual Annual SSNS Meeting (Swiss Society of Neurosurgery), Switzerland (09/17/2022); and as a scientific short-talk at the 14th ESBS Congress (European Skull Base Society Meeting) in Riva del Garda, Italy (04/22/2022).

## Data Availability

The raw data supporting the conclusions of this article will be made available by the authors on reasonable request, without undue reservation.
