# Supplementary material for: Supratentorial cerebrospinal fluid diversion using image-guided trigonal ventriculostomy during retrosigmoid craniotomy for cerebellopontine angle tumors
Source: Front Surg. 2023 May 23;10:1198837. doi: 10.3389/fsurg.2023.1198837 (PMC10242017; doi:10.3389/fsurg.2023.1198837)
Supplement: Supplementary file 1 [file Table1.docx]

| **Univariable analysis** |  | | | |
| --- | --- | --- | --- | --- |
| **Supratentorial hemorrhage** | OR | 95%-CI | | p-value |
|  |  | Lower | Upper |  |
| Age ≥60 years | 1.44 | 0.34 | 6.10 | 0.623 |
| Age (per year) | 1.03 | 0.97 | 1.09 | 0.313 |
| Female sex | 2.77 | 0.52 | 15.0 | 0.235 |
| Posterior fossa volume (cm^3^) | 1.02 | 0.98 | 1.07 | 0.278 |
| Tumor volume (cm^3^) | 1.02 | 0.98 | 1.06 | 0.279 |
| Posterior fossa / tumor volume ratio | 0.96 | 0.88 | 1.03 | 0.250 |
| Preoperative hydrocephalus | 0.74 | 0.13 | 4.07 | 0.727 |
| Preoperative 4^th^ ventricular compression / obstruction | 3.01 | 0.35 | 27.5 | 0.686 |
| Preoperative brainstem compression / dislocation | 5.76 | 0.66 | 50.2 | 0.113 |
| Preoperative cerebellar edema | 0.42 | 0.56 | 3.09 | 0.392 |
| Preoperative tonsillar herniation | 0.77 | 0.08 | 7.32 | 0.821 |
| **Postoperative hydrocephalus** | OR | 95%-CI | | p-value |
|  |  | Lower | Upper |  |
| Age ≥60 years | 1.10 | 0.28 | 4.37 | 0.892 |
| Age (per year) | 0.97 | 0.93 | 1.01 | 0.198 |
| Female sex | 0.11 | 0.21 | 0.60 | 0.011* |
| Posterior fossa volume (cm^3^) | 1.03 | 0.99 | 1.07 | 0.129 |
| Tumor volume (cm^3^) | 0.99 | 0.95 | 1.03 | 0.623 |
| Posterior fossa / tumor volume ratio | 0.99 | 0.93 | 1.04 | 0.693 |
| Preoperative hydrocephalus | 6.36 | 1.45 | 28.0 | 0.014* |
| Preoperative 4^th^ ventricular compression / obstruction | 1.42 | 0.26 | 7.73 | 0.686 |
| Preoperative brainstem compression / dislocation | 1.44 | 0.32 | 6.37 | 0.634 |
| Preoperative cerebellar edema | 1.83 | 0.56 | 5.97 | 0.317 |
| Preoperative tonsillar herniation | 0.67 | 0.07 | 6.26 | 0.723 |
| Ventriculostomy-related hemorrhage | 0.80 | 0.22 | 7.21 | 0.803 |
| **Permanent CSF-diversion after retrosigmoid craniotomy** | OR | 95%-CI | | p-value |
|  |  | Lower | Upper |  |
| Age ≥60 years | 0.24 | 0.25 | 2.31 | 0.216 |
| Age (per year) | 0.93 | 0.88 | 0.99 | 0.020* |
| Female sex | 0.00 | 0.00 | 0.00 | 0.999 |
| Posterior fossa volume (cm^3^) | 1.05 | 0.99 | 1.11 | 0.120 |
| Tumor volume (cm^3^) | 0.99 | 0.94 | 1.05 | 0.822 |
| Posterior fossa / tumor volume ratio | 1.01 | 0.95 | 1.08 | 0.717 |
| Preoperative hydrocephalus | 1.95 | 0.29 | 13.1 | 0.494 |
| Preoperative 4^th^ ventricular compression / obstruction | 1.37 | 0.14 | 13.5 | 0.787 |
| Preoperative brainstem compression / dislocation | 2.48 | 0.26 | 24.0 | 0.432 |
| Preoperative cerebellar edema | 1.68 | 0.37 | 7.55 | 0.500 |
| Preoperative tonsillar herniation | 1.71 | 0.16 | 18.0 | 0.723 |
| Ventriculostomy-related hemorrhage | 0.00 | 0.00 | 0.00 | 0.999 |

*CSF: cerebro-spinal fluid; OR: Odds-ratio; CI: confidence-interval*

**Supplementary Table S1: Univariable covariate logistic regression analysis.**

Univariable covariate logistic regression analysis for any supratentorial hemorrhagic complication (including ventriculostomy-related hemorrhage, intraventricular hemorrhage and intracerebral hemorrhage), for postoperative hydrocephalus and for permanent CSF-diversion in n=52 patients undergoing supratentorial CSF-diversion using image-guided trigonal ventriculostomy during retrosigmoid craniotomy for cerebellopontine angle tumors. The univariable binary logistic regression model includes the following covariates: age ≥60 years (reference level: age <60 years), age (continuous per year), female sex (reference level: male sex), Posterior fossa volume (cm^3^), Tumor volume (cm^3^) and Posterior fossa / tumor volume ratio (continuous), preoperative hydrocephalus (reference level: absence of hydrocephalus), preoperative 4^th^ ventricular compression / obstruction (reference level: absence of 4^th^ ventricular affection), preoperative brainstem compression / dislocation (reference level: absence of brainstem affection), preoperative cerebellar edema (reference level: absence of cerebellar edema), preoperative tonsillar herniation (reference level: absence of tonsillar herniation), and any supratentorial hemorrhagic complication consisting VRH, IVH and/or ICH (reference level: absence of supratentorial hemorrhagic complication); insignificance was set at alpha level of p = .05. Significance is indicated as follows: * (p ≤ .05), ** (p ≤ .01).
